# Natural variations of cold tolerance and temperature acclimation in *Caenorhabditis elegans*

**DOI:** 10.1007/s00360-016-1011-3

**Published:** 2016-06-18

**Authors:** Misaki Okahata, Akane Ohta, Hitomi Mizutani, Yohei Minakuchi, Atsushi Toyoda, Atsushi Kuhara

**Affiliations:** 1 Laboratory of Molecular and Cellular Regulation, Faculty of Science and Engineering and Institute for Integrative Neurobiology, Konan University, 8-9-1 Okamoto, Higashinada-ku, Kobe, 658-8501 Japan; 2 Comparative Genomics Laboratory, National Institute of Genetics, Mishima, Japan

**Keywords:** *Caenorhabditis elegans*, Natural variation, Cold tolerance, Temperature acclimation

## Abstract

Temperature is critical for the survival and proliferation of animals, which must be adapted to cope with environmental temperature changes. In this study, we demonstrated natural variations in the phenotypes of temperature tolerance and temperature acclimation of the nematode *Caenorhabditis elegans*, and we decoded whole genome sequence of six natural variations, which enabled us to map responsible gene polymorphisms onto specific chromosomal regions. The *C. elegans* laboratory strain, N2, survives at 2 °C after cultivation at 15 °C but is unable to survive at 2 °C after cultivation at 20 or 25 °C. This cultivation-temperature-dependent cold tolerance occurs within a few hours after the temperature shift and is termed cold acclimation. We measured the cold tolerance and cold acclimation phenotypes of many natural variants isolated from various areas. CB4854 showed weaker cold tolerance associated with gene polymorphisms on the sex chromosome decoded by whole genome sequencing. Variable cold acclimation phenotypes were exhibited in twelve natural isolates and the large difference was seen between CB4856 and AB1 strains. CB4856, isolated from Hawaii, acclimated slowly to a new temperature, whereas AB1, isolated from Australia, acclimated rapidly. By the whole genome sequencing analysis, two different polymorphisms responsible for the accelerated cold acclimation in AB1 were mapped to specific chromosomal regions.

**Electronic supplementary material:**

The online version of this article (doi:10.1007/s00360-016-1011-3) contains supplementary material, which is available to authorized users.

## Introduction

Temperature is one of the most important environmental factors for organisms. Most animals, including humans, have evolved to cope with warming and cooling and have developed mechanisms for acclimation to and tolerance of temperature changes. The molecular biology of temperature tolerance and acclimation has been analyzed in many organisms. Changes in temperature sensation, metabolic state, and lipid composition are key factors underlying these processes in *Caenorhabditis elegans* (Murray et al. 2007; Ohta et al. 2014; Savory et al. 2011; Xiao et al. 2013).

To gain novel insight into temperature tolerance and acclimation of animals, we performed comparative phenotypic and genetic analyses of a number of wild-type strains of *C. elegans* isolated from different areas. It is known that *C. elegans* has mechanisms to cope with temperature changes, such as forming dauer larvae at high temperatures (Golden and Riddle 1984; Hu 2007) and acquiring tolerance against low temperatures (Murray et al. 2007; Ohta et al. 2014; Savory et al. 2011). In cold tolerance and acclimation, our previous studies described an essential cell network, where temperature is sensed by a head sensory neuron, which releases insulin that acts on the intestine (Ohta et al. 2014).

Cold acclimation of *C. elegans* demonstrates plasticity because a change in cold tolerance occurs after only 2–3 h exposure to a changed cultivation temperature (Ohta et al. 2014). N2 cultured at 25 °C do not survive at 2 °C, whereas N2 cultured at 15 °C do. However, when N2 animals cultured at 25 °C are transferred to 15 °C for 3 h, they are able to survive at 2 °C (Ohta et al. 2014). The molecular physiological mechanisms for this phenomenon are thought to involve fundamental mechanisms of plasticity in metabolic systems and/or memory dynamism in temperature responses.

To investigate the mechanism of cold acclimation, we carried out a comparative analysis among natural wild-type isolates because natural variants have already adapted to their environmental temperature conditions. Previous reports suggest that temperature-dependent phenotypes of wild-type isolates can be divided into several groups. For example, lifetime fecundity or fertility was found to be temperature dependent in wild-type isolates (Harvey and Viney 2007; Petrella 2014), suggesting that natural isolates respond differently to temperature, reflecting genomic variation. We speculated that wild-type isolates from different areas would exhibit different cold tolerance and acclimation phenotypes. Thirteen natural wild-type isolates were obtained from the *C. elegans* Genetics Center (CGC) because these strains were used in an analysis of natural variation in a previous study (de Bono and Bargmann 1998). We measured their phenotypes of cold tolerance after cultivating them at constant temperature, and of cold acclimation after cultivating them with a temperature shift.

Previous reports demonstrated the usefulness of analyzing naturally varying phenotypes to identify novel genes involved in a range of biological traits. Recombinant inbred lines derived from N2 and other wild isolate strains have been used for mapping genes underlying complex traits, including gene expression (Li et al. 2006), lifespan (Doroszuk et al. 2009), and life history traits in stressful environments, such as during dauer formation (Harvey et al. 2008).

Here, we report the genetic analysis of natural variation in cold acclimation. We investigated the variety of phenotypes associated with cultivation-temperature-dependent cold tolerance and acclimation in natural wild-type isolates of *C. elegans*. Several strains, including CB4854, KR314, and AB1, showed differences in cold tolerance or cold acclimation. A possible candidate gene polymorphism responsible for natural variation in cold tolerance in CB4854 was mapped to the sex chromosome, and at least two candidate polymorphisms responsible for cold acclimation in AB1 were mapped onto chromosome I. We decoded whole genome sequences of six wild-type isolates, which will be useful for further analysis of the effects of naturally accumulated polymorphisms.

## Methods

### Strains

We used the following *C. elegans* strains: N2 Bristol England, CB4852 Rothamsted, Harpenden, England; CB4853 Altadena, California, CB4854 Altadena, California; CB4855 Palo Alto, California; CB4856 Hawaii, CB4857 Claremont, California; CB4858 Pasadena, California; CB4932 Taunton, England, AB1 Adelaide, Australia; AB3 Adelaide, Australia, RC301 Freiburg, Germany; and KR314 Vancouver, Canada.

### Statistical analysis

All error bars in the figures indicate the standard error of the mean (SEM). All statistical analyses were performed by one-way analysis of variance for multiple comparisons, followed by Dunnett’s post hoc tests. Single asterisk and double asterisks in the figures indicate *P* < 0.05 and *P* < 0.01, respectively.

### Cold tolerance assay

The cold tolerance assay was performed according to a previously reported protocol (Ujisawa et al. 2014). Uncrowded and well-fed animals were used. Two animals (P0) were placed onto nematode growth medium (NGM) in 60 mm diameter plates and incubated at each experimental temperature for 8–12 h until approximately 70–150 eggs had been laid. The P0 animals were removed after egg laying. CB4853 differed slightly in growth rate compared with the other twelve wild-type isolates, and was, therefore, incubated for 3–5 h longer than the other isolates at 15, 20, 25 °C to adjust for this difference. After incubation at each temperature, plates were directly placed on ice for 20 min and then transferred to a 2 °C refrigerator (CRB-41A, Hitachi, Japan) for 48 h. After 48 h, assay plates were transferred to an incubator at 15 °C. The numbers of live and dead worms were counted on the following day, and the survival rate was calculated. In linkage mapping or three-factor cross experiments, the culture temperature is indicated in the methods or figure legends. The cold tolerance assay was performed on three to nine plates per strain.

### Linkage mapping

Nucleotide polymorphisms in CB4854 responsible for the observed cold tolerance phenotypes were mapped to linkage groups using the following visible marker strains: *dpy*-*5*(*e61*) I, *vab*-*9*(*e1744*) II; *him*-*5*(*e1490*) V, *unc*-*32*(*e189*) III, *unc*-*8*(*e49*) IV, *unc*-*42*(*e270*) V and *lon*-*2*(*e678*) X.

### Three-factor cross

Non-Lon progeny from outcrosses between CB4854 and *lon*-*2*(*e678*) were named CB4854(*lon*-*2 kickout*) and were used for three-factor crosses. CB4854(*lon*-*2 kickout*) showed a low survival rate after culture at 17 °C; this was because they had an X chromosome derived from CB4854, while the other chromosomes were heterozygous. The F1 progeny, CB4854/*lon*-*2 unc*-*84*, was outcrossed with CB4854(*lon*-*2 kickout*) and *lon*-*2*(*e678*)*; unc*-*84*(*e1410*). From CB4854/*lon*-*2 unc*-*84* hermaphrodites, 11 of 43 Lon Non-Unc recombinants segregated CB4854, and two of 14 Unc Non-Lon recombinants segregated CB4854. Because the responsible polymorphism might be located between *lon*-*2* and *unc*-*84*, we used the Lon Non-Unc recombinant (named KHR046), which showed a low cold tolerance survival rate after culture at 15 °C, for the next three-factor cross. In this cross, KHR046, the Lon Non-Unc recombinant, was crossed with *unc*-*84*(*e1410*), and 59 recombinants (Lon Unc) were obtained and their cold tolerance (F2) phenotypes were examined. Thirteen of the 59 Lon Unc recombinants showed a low cold tolerance phenotype after 15 °C culture, while the other recombinants showed similar survival rates as N2.

### Cold acclimation assay

*Caenorhabditis elegans* were cultured under well-fed conditions. Three or more well-fed adults (P0) were placed on a 3.5 cm NGM with 2 % (w/v) agar plate at the first temperature and incubated until they had laid ~100 eggs (≥3 h). P0 adults were then removed to synchronize the growth stages of the progeny.

Progeny were incubated from egg to adult at the initial temperature. For example, progeny were cultured for 132–150 h at 15 °C, or cultured for 52–65 h at 25 °C. When the animals grew to young adults or adults, the assay plates were transferred to a second temperature for several hours (e.g., 0, 3, 5, 8, or 18 h). For transfer to the second temperature for 18 h, L4 larvae were used. After incubation at the second temperature, plates were directly placed on ice for 20 min and then transferred to a 2 °C refrigerator (CRB-41A, Hitachi, Japan) for 48 h. After 48 h, assay plates were incubated at 15 °C. The numbers of live and dead worms were counted on the following day. Each cold acclimation assay was performed three times with more than nine plates.

### Whole genome sequencing

Worms for genome extraction were cultured in NGM under well-fed conditions at 20 °C. High quality genomic DNA was extracted using a Gentra Puregene Tissue Kit (QIAGEN, Hilden, Germany) according to the manufacturer’s instructions. The genome sequences of KR314, RC301, CB4854, and N2 were determined on an Illumina Genome Analyzer IIx platform using an Illumina TruSeq DNA Sample Prep Kit (Illumina, San Diego, CA, USA), which generated from 100 to 140 million reads; each read length was 110 bp. The genome sequences of AB1, CB4852, CB4853 were determined on an Illumina Hiseq 2500 platform using a TruSeq DNA PCR-Free LT Sample Prep Kit (Illumina), which generated from 50 to 130 million reads; each read length was 150 bp. Over 90 % of the genome was sequenced at a 10X coverage, except for CB4853 in which about 30 % was sequenced at 10X coverage. The nucleotide sequences reported in this paper have been submitted to the DDBJ Sequence Read Archive under accession numbers DRA002599 and DRA004249.

### SNP calling and annotation

Illumina reads were mapped to the *C. elegans* reference genome (UCSC ce10) by BWA v0.6.2 (Li and Durbin 2009) using the default parameters. PCR or optical duplicates were removed using Picard v1.77, and base quality recalibration and realignment around indels were performed by GATK v2.1.8 (DePristo et al. 2011; McKenna et al. 2010). In addition to our sequence data, we used re-sequenced data of CB4856 (Accession Number; SRR443373) to compare AB1 and CB4856.

Variants were called by Unified Genotyper and filtered by Variant Filtration in GATK. The parameters “-strand_call_conf 30.0, -strand_emit_conf 10, -dcov 200” and “–clusterWindowSize 10, –filterExpression ‘(MP0/(1.0*DP)) > 0.05′, –filterName ‘HARD_TO_VALIDATE’, –filterExpression ‘QUAL < 30 || DP < 10 || SB > −0.1 || MQ < 25.0′, –filterName ‘STD_Filter’” were used for Unified Genotyper and Variant Filtration, respectively.

We categorized called variants using SnpEff (Cingolani et al. 2012) into four Impact groups: High, Moderate, Low and Modifier. ‘High’ includes splice_site_acceptor, splice_site_donor, start_lost, exon_deleted, frame_shift, stop_gained, stop_lost, and rare_amino_acid. “Moderate” includes non_synonymous_coding, codon_change, codon_insertion, codon_change_plus_codon_insertion, codon_deletion, codon_change_plus_codon_deletion, UTR_5_deleted and UTR_3_deleted. “Low” includes synonymous_start, non_synonymous_start, start_gained, synonymous_coding and synonymous_stop. “Modifier” includes UTR_5_prime, UTR_3_prime, regulation, upstream, downstream, gene, transcript, exon, intron_conserved, intron, intragenic, intergenic, intergenic_conserved, none, chromosome, custom and cds.

### Mapping of responsible polymorphisms in AB1

We utilized the large phenotypic discrepancy between AB1 from Australia and CB4856 from Hawaii to identify the responsible polymorphism of AB1. We made AB1*; Ex* [pKDK66 *ges*-*1p::nls*-*gfp, AIYp::gfp*] and crossed it with CB4856. F1 heterozygotes were placed on fresh NGM and incubated from egg to L4 or early adult. The animals expressing the GFP marker were isolated as F2, resulting in 71 recombinant inbred (RI) lines. We investigated the phenotypes of these RI lines from F3 to F10. Some of the RI lines showed an AB1-type phenotype, accelerated cold acclimation. The genotypes of these RI lines were analyzed by the SNPs that showed differences between AB1 and CB4856 (Supplementary Table 1). Genotypes were determined by SNP analysis according to a restriction fragment length polymorphism (RFLP) method (Wicks et al. 2001) or by direct sequencing. The primers used for RFLP and direct sequencing are described in Supplementary Fig. 3a.

## Results

### Natural variation of cold tolerance in wild-type *C. elegans*

**Fig. 1:**
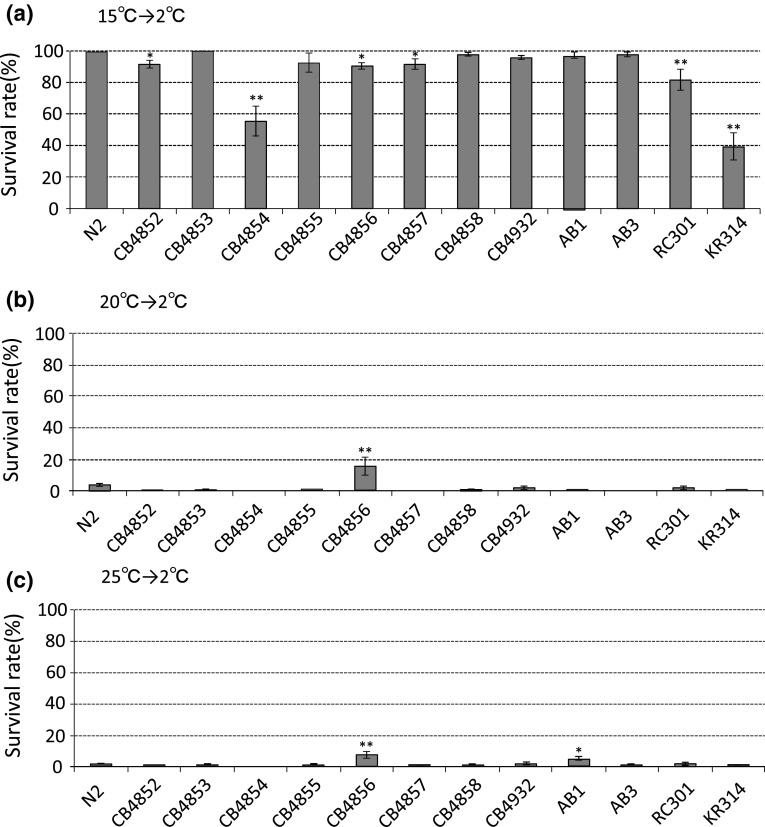
Cold tolerance phenotypes of thirteen natural wild-type isolates from various countries. The cold tolerance phenotypes of thirteen wild-type isolates were measured when worms were cultivated at 15 °C (**a**), 20 °C (**b**) or 25 °C (**c**) and exposed to a cold shock of 2 °C for 48 h. N2 from Bristol and nine other isolates (CB4852, CB4853, CB4855, CB4856, CB4857, CB4858, CB4932, AB1, and AB3) survive cold shock after cultivation at 15 °C. The survival rates of CB4854, RC301 and KR314 after cultivation at 15 °C were 56, 82, and 39 %, respectively. After cultivation at 20 or 25 °C, only the survival rate of CB4856 was significantly higher than that of the other isolates. For each assay, *n* ≥ 3. *Error bars* indicate SEM. Statistical analyses were performed by one-way analysis of variance for multiple comparisons, followed by Dunnett’s post hoc tests. *Asterisks* indicate statistical significance between the N2 Bristol strain and the other wild-type isolates. *P < 0.05 and **P < 0.01

*Caenorhabditis elegans* exhibits cultivation-temperature-dependent cold tolerance. N2 Bristol animals cultivated at 25 °C do not survive at 2 °C. In contrast, N2 animals cultivated at 15 °C can survive at 2 °C (Ohta et al. 2014). We investigated cold tolerance phenotypes in thirteen natural wild-type isolates from various countries (Fig. 1). After cultivation at 15 °C, the survival rate of the N2 strain was around 100 %, and nine natural wild-type isolates, CB4852, CB4853, CB4855, CB4856, CB4857, CB4858, CB4932, AB1, and AB3, showed similar phenotypes to that of N2 (Fig. 1a). This suggests that these ten wild-type isolates have phenotypically similar cold tolerance at 2 °C, after cultivation at 15 °C. In contrast, the survival rates of CB4854, RC301, and KR314 were significantly lower than the other wild-type isolates. (Fig 1a). These results indicate that polymorphisms of CB4854, RC301, and KR314 cause a decrease in cold tolerance after cultivation at 15 °C. After cultivation at 20 or 25 °C, the survival rates of all strains except for CB4856 were lower than 5 % (Fig. 1b, c). In contrast, the survival rate of CB4856 after 20 °C cultivation was 16 %, which was statistically different from the other wild-type isolates (Fig. 1b, c). These data suggest that polymorphisms of CB4856 slightly increase the survival rate in the cold tolerance test after cultivation at 20 or 25 °C. We previously reported more than a dozen mutants that showed increased survival rates after cultivation at 20 or 25 °C (Ohta et al. 2014); however, mutations causing a decrease in survival rate after 15 °C cultivation are poorly understood.

### Linkage mapping of DNA polymorphisms causing cold tolerance variation

To identify the polymorphisms responsible for the differences in cold tolerance, we needed to identify nucleotide polymorphisms over the whole genome for use as genetic mapping tools. We sequenced the genomes of three strains, CB4854, KR314, and RC301, and identified 79,002, 95,506, and 67,480 SNPs, respectively, compared with N2 (Table 1). Among these polymorphisms, 778, 1004, and 722 in CB4854, KR314, and RC301, respectively, induced a severe effect, categorized as ‘high’, e.g., frame-shift, stop-lost, or stop-gained (see “Methods”) (Supplementary Data 1).

**Table 1:** Summary of the polymorphisms of KR314, RC301, CB4854, AB1, CB4852, and CB4853

	Homo		Hetero	
	SNP	Indel	SNP	Indel
N2	1129	1779	1167	165
KR314	95,506	37,903	10,130	2718
RC301	67,480	26,750	7557	1976
CB4854	79,002	32,353	9213	2582
AB1	66,710	28,213	16,267	4553
CB4852	50,458	21,540	12,964	3300
CB4853	75,951	29,330	12,192	4060

SNPs and insertions or deletions (indels) were called using Unified Genotyper in the Genome Analysis Toolkit (GATK). The numbers of SNPs in other strains were identified by comparison with the N2 sequence database (*C. elegans* UCSC ce10). For example, our whole genome sequencing analysis identified 95,506 putative SNPs in KR314 relative to N2. We also sequenced the whole N2 genome (N2 Kuhara lab) using N2 worms maintained in our laboratory and compared it with N2 of UCSC ce10

**Fig. 2:**
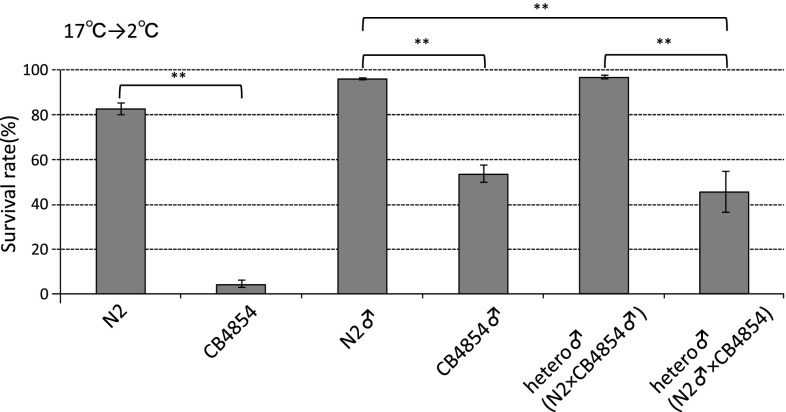
Cold tolerance phenotypes of heterozygous males. A significant difference of survival rate was observed between N2 hermaphrodites (indicated as N2) and CB4854 hermaphrodites (indicated as CB4854) after cultivation at 17 °C. The survival rates of homozygous N2 males (indicated as N2♂) and homozygous CB4854 males (indicated as CB4854♂) were significantly different. From analysis of the effect of autosomal heterozygosity and hemizygosity of the sex chromosome, heterozygous males (indicated as N2xCB4854♂) outcrossed from N2 hermaphrodites and CB4854 males showed high survival rates, similar to homozygous N2 males. In contrast, the survival rate of other heterozygous males (indicated as N2♂xCB4854) outcrossed from CB4854 hermaphrodites and N2 males was decreased to the same level as homozygous CB4854 males. Therefore, the two heterozygous males showed different cold tolerance phenotypes. These data suggest that the CB4854 X chromosome contains the polymorphism responsible for cold tolerance. For each assay, *n* ≥ 9. *Error bars* indicate SEM. Statistical analyses were performed by one-way analysis of variance for multiple comparisons, followed by Dunnett’s post hoc tests. **P < 0.01

Because only CB4854 showed a significantly decreased survival rate at 2 °C following culture at 15 °C, and also at 17 °C (Supplementary Fig. 1), we tried to identify the polymorphisms responsible for the reduced cold tolerance in CB4854, by classical genetic mapping methods. The results of linkage mapping indicated that the responsible polymorphisms were located on the X chromosome (see “Methods”). To confirm these results, we investigated cold tolerance phenotypes of males that were outcrossed between N2 and CB4854 (Fig. 2). Heterozygote males (N2-type/CB4854-type [autosome]; N2-type/ [sex chromosome]) outcrossed from N2 hermaphrodites and CB4854 males showed the cold-tolerant phenotype. However, other heterozygous males (N2-type/CB4854-type [autosome]; CB4854-type/ [sex chromosome]) outcrossed from CB4854 hermaphrodites and N2 males showed significantly lower survival rates than N2-type males whose sex chromosome was derived from N2 (Fig. 2). These data support an X chromosome-linked CB4854 genotype causing decreased cold tolerance.

**Fig. 3:**
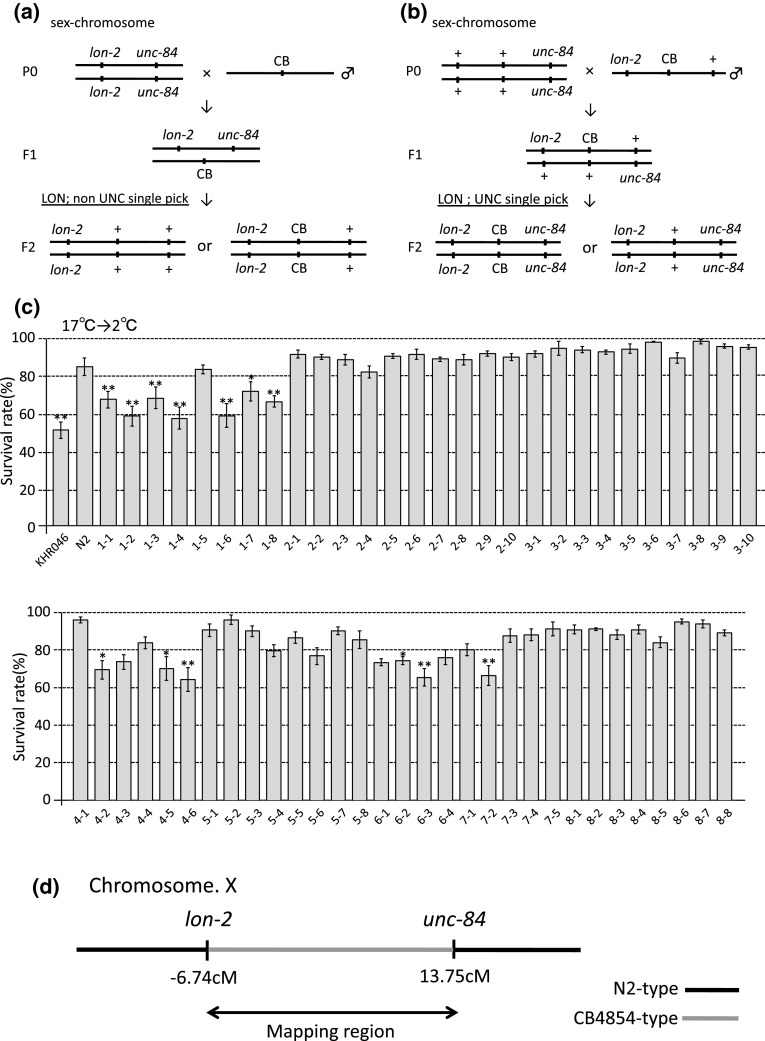
Three-factor cross to identify the chromosomal region containing the polymorphism responsible for the CB4854 phenotype. We performed three-factor crosses in twice, as indicated in the sex chromosome schemes in panels (**a**), (**b**). CB in **a**, **b** indicate a putative polymorphism responsible for natural variation of cold tolerance in CB4854. **a** The double mutant hermaphrodite *lon*-*2*(*e678*)*; unc*-*84*(*e1410*) was crossed with male CB4854(*lon*-*2 kickout*). CB4854(*lon*-*2 kickout*) is the previously obtained progeny from an outcross between CB4854 and *lon*-*2*(*e678*) that showed Non-Lon in the linkage mapping, and low cold tolerance similar to CB4854. From CB4854/*lon*-*2 unc*-*84* hermaphrodites (F1), we obtained Lon Non-Unc recombinants (F2) that showed a low survival rate after cultivation at 17 °C, named KHR046. **b** The Lon Non-Unc recombinants, KHR046 in **c**, were crossed with *unc*-*84*(*e1410*) (P0). 59 recombinants (Lon Unc) were obtained (F2) and their phenotypes of cold tolerance examined. **c** The cold tolerance phenotypes of 59 recombinants from the second three-factor cross. The recombinants indicated as 1-1, 1-2, 1-3, 1-4, 1-6, 1-7, 1-8, 4-2, 4-5, 4-6, 6-2, 6-3, and 7-2 showed significantly lower survival rates than that of N2 and the other recombinants showed high survival rates, similar to N2. These data suggest that the recombinants showing a low cold tolerance phenotype have the polymorphism responsible, and that the other strains do not have that polymorphism between *lon*-*2* and *unc*-*84*. For each assay, *n* ≥ 3. *Error bars* indicate SEM. Statistical analyses were performed by one-way analysis of variance for multiple comparisons, followed by Dunnett’s post hoc tests. *Asterisks* indicate significant differences between the N2 Bristol strain and recombinants. *P < 0.05 and **P < 0.01. **d** The polymorphism responsible for low cold tolerance in CB4854 was mapped between *lon*-*2* (−6.74 cM) and *unc*-*84* (13.75 cM)

We performed a three-factor cross using *lon*-*2*(-6.74 cM) and *unc*-*84*(13.75 cM), both genes located on the X chromosome. From CB/*lon*-*2 unc*-*84* hermaphrodites (F1 as indicated in Fig. 3a) we obtained Lon non-Unc recombinants (Fig. 3a) that showed a low survival rate after cultivation at 17 °C (Fig. 3c), and we named one strain of the recombinants KHR046. KHR046 was crossed with *unc*-*84*(*e1410*), and 59 recombinants (Lon Unc) were obtained and used to determine their cold tolerance phenotypes (Fig. 3b). Thirteen of the 59 recombinants showed statistically lower survival rates than that of the N2 strain (Fig. 3c). These data suggest that one of the polymorphisms decreasing survival rate in KHR046 is located between *lon*-*2* and *unc*-*84* genes on the sex chromosome (Fig. 3d). However, the survival rates of KHR046 and other recombinants in cold tolerance tests after cultivating at 17 °C were greater than 50 %, and significantly higher than that of the original CB4854 strain (Supplementary Fig. 1). We hypothesized that the other polymorphism(s) contribute to the decrement of survival rate in CB4854. The survival rate of KHR046 was elevated to greater than 50 % because KHR046 (LON non-UNC) had lost other polymorphism(s) through recombination, as indicated in Fig. 3a.

We next investigated the genotypes of the following recombinants: 1-2, 1-8, 4-6, 6-1, 3-8, and 8-2. These recombinants can be grouped by their survival rates into a lower group (1-2, 1-8, 4-6, and 6-1) and a higher group (3-8 and 8-2). Investigation of genotypes at about ten loci between −2.9 and 5.15 cM revealed that CB4854-type SNPs were located between −1.6 and 2.2 cM in both the lower and higher groups (Supplementary Fig. 2). This apparent discrepancy between the phenotypes and genotypes indicated that identification of one possible polymorphism decreasing cold tolerance of KHR046 was difficult using these recombinants.

### Natural variation in the rate of cold acclimation in wild-type *C. elegans*

**Fig. 4:**
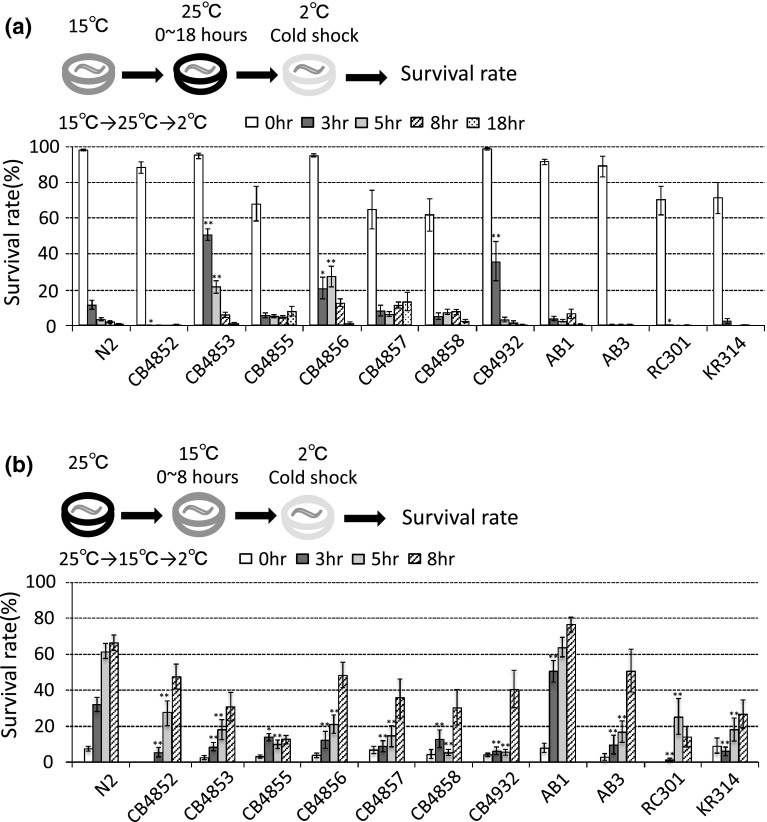
Temperature acclimation in natural wild-type isolates. **a** Twelve natural variation strains were subjected to the cold acclimation test. 15 °C cultivated animals were transferred to 25 °C for 0, 3, 5, 8, or 18 h. The survival rate of the N2 strain at 15 °C cultivation with no incubation at 25 °C (0 h) was 98 %. From 60 % to 100 % of animals in other strains survive at 2 °C after cultivation at 15 °C with no 25 °C incubation (*white bars*). When the animals were incubated for a few hours at 25 °C after cultivation at 15 °C, the survival rate decreased significantly, suggesting that animals acclimate to the new temperature within a few hours. For example, the survival rate of N2 from Bristol decreased to 12 and 4 % after 3 and 5 h culture at 25 °C, respectively. Five strains, CB4852, AB1, AB3, RC301, and KR314 acclimated to 25 °C in 3 h, which was faster than for N2. Fifty-one percent of CB4853, 21 % of CB4856 and 36 % of CB4932 survived at 2 °C, even after cultivation at 25 °C for 3 h. For each assay, *n* ≥ 6. *Error bars* indicate SEM. Statistical analyses were performed by one-way analysis of variance for multiple comparisons, followed by Dunnett’s post hoc tests. *Asterisks* indicate significant differences between the N2 Bristol strain and the other wild-type isolates with the same duration of 25 °C incubation. *P < 0.05 and **P < 0.01. **b** Animals cultivated at 25 °C were transferred to 15 °C and incubated for 0, 3, 5, or 8 h. The survival rates of N2 incubated for 3 or 5 h at 15 °C were 32 and 62 %. Moreover, about 50 % of AB1 from Adelaide survived after only 3 h incubation at 15 °C. These data suggest that the AB1 and N2 strains acclimated faster to 15 °C than the other ten strains did. After incubation at 15 °C for 8 h, the survival rates of CB4852, CB4856 and AB3 reached 50 %, but the rates of the other seven strains remained below 40 %. For each assay, *n* ≥ 6. *Error bars* indicate SEM. Statistical analyses were performed by one-way analysis of variance for multiple comparisons, followed by Dunnett’s post hoc tests. *Asterisks* indicate significant differences between the N2 Bristol strain and the other wild-type isolates with the same duration of 15 °C incubation. *P < 0.05 and **P < 0.01

We investigated whether natural wild-type isolates differed in their rates of temperature acclimation. The rate of temperature acclimation was determined from the time course of the cold acclimation assay. Larvae or young adults were grown at 15 °C and then cultivated at 25 °C for the designated time (0, 3, 5, 8, or 18 h). They were then subjected to the 2 °C cold stimulus. We measured the temperature acclimation rate of twelve natural wild-type isolates (Fig. 4a). The survival rate of N2 Bristol was strongly reduced after incubation at 25 °C for 3 and 5 h following cultivate at 15 °C (Fig. 4a). These data suggest that the N2 strain acclimates to 25 °C within 3 h. Seven strains (CB4852, CB4855, CB4858, AB1, AB3, RC301, and KR314) acclimated to 25 °C within 5 h; i.e., their 2 °C survival rates after 5 h of incubation at 25 °C were reduced to less than 10 % (Fig. 4a). In addition, significantly lower survival rates were observed for CB4852, AB1, AB3, RC301, and KR314 incubated at 15 °C then at 25 °C for only 3 h. In contrast, the survival rates of CB4853 were higher than that of N2 after 3 and 5 h incubation (Fig. 4a). The survival rates of CB4856 after 5 h incubation at 25 °C and CB4932 after 3 h incubation were also high (Fig. 4a). These observations suggest that rates of acclimation from 15 to 25 °C for CB4852, AB1, AB3, RC301, and KR314 are faster than that of N2. Conversely, the acclimation rates of CB4853, CB4856, and CB4932 were slower than that of N2.

We also measured twelve isolates for the inverse time course test, i.e., from 25 to 15 °C. In the temperature shift from 25 to 15 °C, about 60 % of N2 and AB1 animals survived after only 5 h incubation at 15 °C (Fig. 4b). These data suggest that N2 and AB1 strains can acclimate to 15 °C within 3 or 5 h. Approximately 50 % of CB4852, CB4856, and AB3 survived after incubation at 15 °C for 8 h; however, the survival rates of the other seven strains—CB4853, CB4855, CB4857, CB4858, CB4932, RC301, and KR314—remained below 40 % (Fig. 4b). These results suggest that either these ten strains need at least 8 h to acclimate to 15 °C after transfer from 25 °C, or that over half the population cannot acclimate to 15 °C after cultivation at 25 °C.

### Genetic mapping of DNA polymorphisms responsible for variation of cold acclimation rate

There were differences among the natural wild-type isolates in the characteristics of temperature acclimation. Therefore, we next used genetic analysis to identify the resposible polymorphisms. First, we decoded the whole genome sequences of CB4852, CB4853, and AB1 (Accession Number; DRA004249). We identified 50,458, 75,951, and 66,710 polymorphisms in CB4852, CB4853 and AB1, respectively, compared with N2 (Table 1). Among these, 559, 766, and 786 polymorphisms, respectively, were considered to have a severe effect (categorized as ‘high’ in the “Methods”) (Table 2). Compared with CB4856 from Hawaii, AB1 from Australia had 3489 polymorphisms in coding regions in the high and moderate categories (see Supplementary Table 2 and Supplementary Data 3). Compared with CB4856, the numbers of AB1 polymorphisms with high and moderate effect on chromosomes I, II, III, IV, V, and X, were 319, 746, 574, 540, 945, and 365, respectively (Supplementary Table 2). These whole genome sequences of the three strains are a valuable resource for genetic mapping and SNP analysis using RFLP.

**Table 2:** Summary of polymorphisms called by comparing AB1, CB4852 and CB4853 against N2

Impact	Effect	AB1		CB4852		CB4853	
		Homo	Hetero	Homo	Hetero	Homo	Hetero
High	Splice_site_acceptor	47	7	23	8	45	5
	Splice_site_donor	54	13	34	7	69	10
	Start_lost	8	3	6	5	11	3
	Exon_deleted	0	0	0	0	0	0
	Frame_shift	549	85	392	80	516	105
	Stop_gained	98	18	80	21	96	17
	Stop_lost	30	3	24	1	29	1
	Rare_amino_acid	0	0	0	0	0	0
	Total	786	129	559	122	766	141
Moderate	Non_synonymous_coding	6531	781	4941	728	8501	682
	Codon_change	0	0	0	0	0	0
	Codon_insertion	86	14	73	3	87	14
	Codon_change_plus_codon_insertion	48	4	33	4	48	9
	Codon_deletion	92	9	64	8	130	28
	Codon_change_plus_codon_deletion	71	10	42	4	61	17
	UTR_5_deleted	0	0	0	0	0	0
	UTR_3_deleted	0	0	0	0	0	0
	Total	6828	818	5153	747	8827	750
Low	Synonymous_start	0	0	0	0	0	0
	Non_synonymous_start	0	0	1	0	1	0
	Start_gained	114	3	80	6	118	2
	Synonymous_coding	7991	636	5378	577	8814	534
	Synonymous_stop	13	0	7	3	11	0
	Total	8118	639	5466	586	8944	536
Modifier	UTR_5_prime	821	76	689	42	944	43
	UTR_3_prime	2794	333	2178	263	3385	224
	Regulation	0	0	0	0	0	0
	Upstream	182,897	37,510	133,808	30,144	196,252	29,675
	Downstream	182,267	40,186	130,353	31,281	190,429	31,273
	Gene	0	0	0	0	0	0
	Transcript	0	0	0	0	0	0
	Exon	1668	469	1343	416	1762	478
	Intron_conserved	0	0	0	0	0	0
	Intron	76,787	18,202	56,441	14,240	87,299	14,447
	Intragenic	0	8	0	7	0	3
	Intergenic	984	320	752	238	930	189
	Intergenic_conserved	0	0	0	0	0	0
	None	0	0	0	0	0	0
	Chromosome	0	0	0	0	0	0
	Custom	0	0	0	0	0	0
	CDS	0	0	0	0	0	0
	Total	448,218	97,104	325,564	76,631	481,001	76,332

The number of polymorphisms identified in AB1, CB4852 and CB4853 against N2 by the GATK Unified Genotyper and Variant Filtration. The N2 sequence data obtained in this study (indicated in Table 1) was used for this analysis. Impact is categorized into high, moderate, low, and modifier according to the effect of the polymorphism (see SNP calling in “Methods”)

**Fig. 5:**
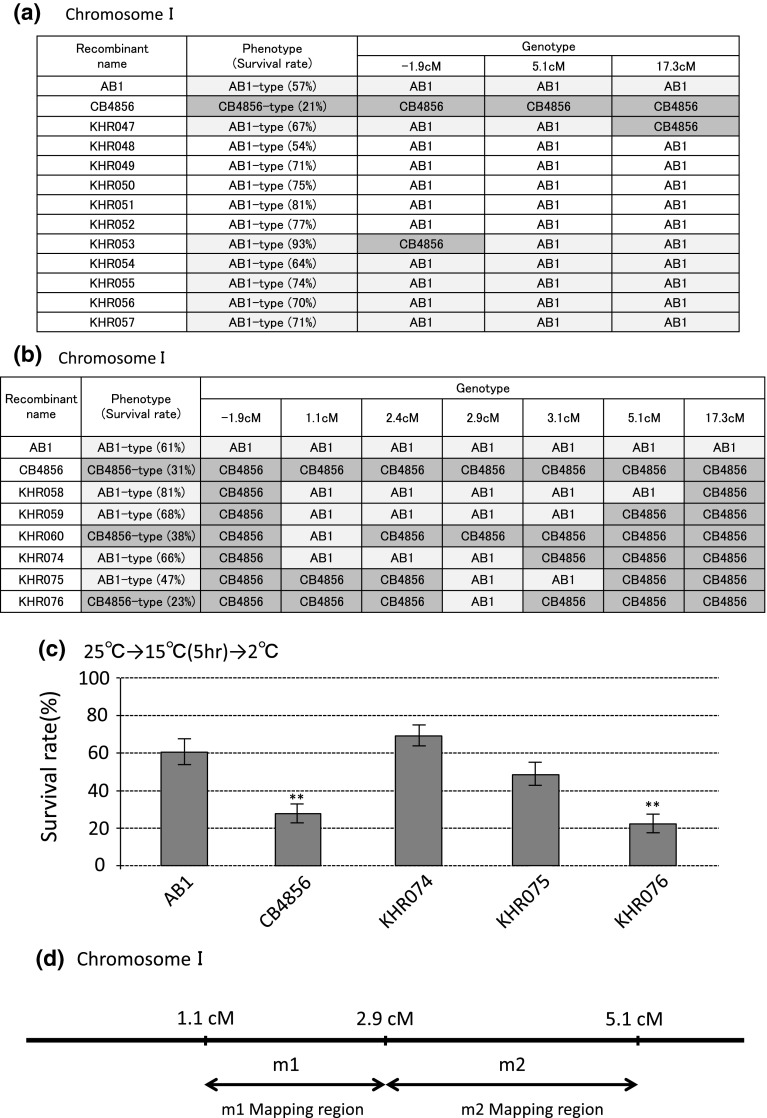
Genotypes and phenotypes of representative recombinant inbred lines for identification of the polymorphisms responsible for the AB1 phenotype. **a** Representatives of recombinant inbred (RI) lines generated between AB1 and CB4856. Cold acclimation phenotypes were determined by using the temperature shift assay of 25–15 °C, with incubation for 5 h. The *numbers* in *parentheses* in the phenotype columns are the survival rates following cold acclimation. Phenotypes AB1-type and CB4856-type were determined from their survival rates. Among 71 strains, 11 RI lines, KHR047–KHR057, showed survival rates greater than 50 %, similar to the AB1 strain. These 11 RI lines showed the AB1-type genotype for the SNP at 5.1 cM on chromosome I. In addition, the genotype of recombinant KHR047 at 17.3 cM and the genotype of KHR053 at −1.9 cM were CB4856-type. **b**, **c** Genotype and cold acclimation phenotype of representative backcrossed recombinant lines. **d** Genomic *scheme* indicating the region mapped with two polymorphisms ‘m1’ and ‘m2’ responsible for fast cold acclimation. m1 responsible polymorphism causing KHR074 phenotype, m2 responsible polymorphism causing KHR075

To identify the polymorphisms responsible for the differences in cold acclimation, we utilized the large difference in cold acclimation rate between AB1 and CB4856 (Fig. 4b). We made recombinant inbred (RI) lines by crossing AB1 with CB4856 and isolated 71 RI lines. The whole genome sequence of CB4856 is publicly available (Wicks et al. 2001), and we were easily able to examine SNPs using RFLP between N2 and CB4856. We used re-sequenced data of CB4856 (Accession Number; SRR443373) and confirmed that many SNPs are distinguishable between AB1 and CB4856 by comparing their genome sequences (Supplementary Tables 1 and 2). We investigated the phenotypes of temperature acclimation and the genotypes of SNPs for the RI lines. Eleven RI lines showed high survival rates similar to AB1 (i.e., rapid cold acclimation) (Fig. 5a) and 17 RI lines showed a low survival rate similar to CB4856 (i.e., slow cold acclimation). We investigated genotypes at three SNPs on each chromosome using RFLP methods in the 11 rapid acclimating RI lines. Analysis with pooled DNA of the 11 RI lines indicated that these lines consist of both AB1-type and CB4856-type SNPs on chromosomes II, III, IV, V, and X (sex chromosome), however, at 5.1 cM on chromosome I, the 11 RI lines consisted of wholly AB1-type SNPs. We next determined the genotypes of the 11 individual RI lines on chromosome I; likewise the genotypes of almost of the lines were AB1-type (Fig. 5a). However, KHR047 showed a CB4856-type SNP at 17.3 cM and the KHR053 showed a CB4856-type SNP at −1.9 cM (Fig. 5a). Therefore, a polymorphism(s) responsible for rapid cold acclimation is located between −1.9 and 17.3 cM on chromosome I.

We also tried to map a polymorphism responsible for the CB4856 phenotype by performing SNP analysis of the 17 slowly acclimating RI lines. We investigated genotypes of the 17 RI lines at three points on each chromosome. However, both the AB1-type genotype and the CB4856-type genotype were detected in the 17 RI lines at all points, implying that CB4856 may have multiple gene polymorphisms involved in temperature acclimation. A polymorphism responsible for the CB4856 phenotype was not mapped onto a single locus by crossing with AB1 (data not shown).

We then obtained other recombinant lines by three times outcrossing with RI lines and CB4856 to narrow down the region of the AB1 genome on chromosome I. From the first crossing, the recombinant outcrossed with KHR047 and KHR053 was backcrossed with CB4856 and we obtained backcrossed RI lines named KHR058, KHR059, KHR060, and KHR074 (Fig. 5b, c). The cold acclimation test with temperature shift from 25 to 15 °C for 5 h indicated that KHR058, KHR059, and KHR074 rapidly acclimated to 15 °C, similar to AB1, and that KHR060 slowly acclimated to 15 °C, similar to CB4856. Genotype analysis using SNPs revealed that the genotypes at chromosome II–V and X of KHR058, KHR059, KHR060, and KHR074 were replaced by the CB4856-type. The genotypes of chromosome I in these four recombinants are shown in Fig. 5b. The common region of the rapidly cold acclimating lines was from −1.9 to 3.1 cM. Moreover, considering that slowly acclimating KHR060 had lost the AB1 genome, at least from 1.1 to 5.1 cM, we hypothesized that the polymorphism responsible for rapid cold acclimation is located in the region from 1.1 to 5.1 cM.

Secondly, we obtained another RI line, KHR075, by outcrossing KHR059 and CB4856. KHR075 showed a relatively high survival rate in the cold acclimation test (Fig. 5c). The AB1 genomic region of KHR075 was narrowed down to between 2.4 cM and 5.1 cM (Fig. 5b). Finally, the recombinant KHR076 was made by outcrossing with KHR075 and KHR074; KHR076 possesses the AB1 genome only in the region from 2.4 to 3.1 cM. Unexpectedly, the survival rate of KHR076 in the cold acclimation test was significantly decreased from that of its parental RI lines, KHR075 and KHR074 (Fig. 5c). These observations suggest that the polymorphisms responsible for rapid cold acclimation are located where KHR076 has lost the AB1 genome, specifically in two separate regions: from 1.1 to 2.9 cM and from 2.9 to 5.1 cM (Fig. 5d).

## Discussion

Elucidating temperature acclimation systems is important in the understanding of environmental adaptability against global climate change. The results in this study show that natural wild-type isolates of *C. elegans* exhibit a range of temperature tolerance and temperature acclimation rates. At least two candidate polymorphisms responsible for cold acclimation in AB1 were mapped between 1.1 and 5.1 cM on chromosome I (Fig. 5d). The whole genome sequences determined for six strains will be useful for the future comparison of natural wild-type isolates.

### Variation of cold tolerance in a variety of natural isolates

In the cold tolerance test, the characteristic phenotypes after cultivation at 15 °C were identified in CB4854 from Altadena in California, RC301 from Freiburg and KR314 from Vancouver (Fig. 1a). These three strains were isolated from locations with different profiles of latitude and climatic conditions. The weak cold tolerance in those strains could be caused by polymorphisms adverse to cold tolerance such as the capacity to survive 2 °C after 15 °C cultivation. Polymorphisms adverse to cold tolerance might have been accumulated over numerous generations living in those areas. In contrast, there is a counterexample. CB4853 from Altadena and CB4858 from Pasadena were isolated areas neighboring that of CB4854, however, CB4853 and CB4858 were able to survive at 2 °C after cultivation at 15 °C, whose phenotypes are different from that of CB4854. This suggests that a significant relationship between cold tolerance and climatic conditions does not exist. Thus, the factors leading to accumulation of polymorphisms in these natural isolates could be not only ambient temperature but also other environmental triggers. The accumulations of a number of polymorphisms are possibly caused by a trade-off between cold tolerance and genetic traits, which is needed for life and reproduction. Although we periodically thaw the strain stock tubes from deep freezer to avoid culturing dozens generations during our investigations into the phenotypes of natural strains, these strains have also been cultivated in standard lab conditions in other laboratories before sending to *C. elegans* stock center, it is possible that spontaneous mutation(s) altered some of their adaptive properties.

A low cold tolerance after cultivation at 15 °C, as shown for three isolates, was not often detected in our previous study using dozens of mutant strains (Ohta et al. 2014). We tried to identify genes responsible for reduced cold tolerance but this was unsuccessful, possibly because it involves complex traits caused by multiple polymorphisms on the sex chromosome. One possible polymorphism was located between −1.65 and 3.52 cM on the sex chromosome but its phenotype was not strong enough to distinguish whether the survival rate was reduced in the recombinants (Fig. 3b). Thus, we needed to produce recombinants in which CB4854 SNPs are located on two different chromosomal regions: from *lon*-*2* (or −1.6 cM) to the left end of the sex chromosome, and from −1.65 and 3.52 cM. Our previous study using the N2 strain revealed that temperature is sensed by the ASJ sensory neuron in the head and that insulin secreted from ASJ controls cold tolerance status (Ohta et al. 2014). The downstream insulin signaling regulating cold tolerance remains unknown. Identification of the genes responsible for the CB4854 phenotype may provide novel insight into the molecular mechanisms of cold tolerance.

The survival rates at 2 °C for CB4856 after cultivation at 20 or 25 °C were statistically different from the other wild-type isolates (Fig. 1b, c). We confirmed whether CB4856 has polymorphisms in genes involved in temperature tolerance in a previous study (Ohta et al. 2014). There were missense mutations of five genes in the CB4856 genome: *daf*-*11, age*-*1, cgt*-*1, dmd*-*7,* and *M60.2*. Although null mutants of these genes showed 40–80 % survival rates at 2 °C after cultivation at 20 °C (Ohta et al. 2014), the survival rates of CB4856 in cold tolerance tests after 20 and 25 °C were 16 and 7.5 %, respectively (Fig. 1b, c), which are much lower than in the knockout mutants. We consider two possibilities: (1) polymorphisms in previously identified cold tolerance genes in CB4856 weakly affect temperature tolerance, although these are not null mutations; or (2) polymorphisms in unidentified genes weakly affect cold tolerance in CB4856.

### Differences of cold acclimation in natural variants

The cold acclimation assay is capable of measuring the rate of acclimation to a new temperature. In a temperature shift from 25 to 15 °C (and from 15 to 25 °C), the acclimation rate was variable among the different strains (Fig. 4a, b). We investigated cold acclimation phenotypes in inverse protocols: from 25 to 15 °C and from 15 to 25 °C. The CB4853 strain slowly acclimated to the second temperature, whereas the AB1 strain rapidly acclimated to the second temperature. These results suggest that common positive and negative regulators act in both temperature shift conditions. Against this hypothesis, the cold acclimation rate from 25 to 15 °C was slower for CB4852 than for the other natural variant strains, but was faster than other strains, including N2, for 15 to 25 °C (Fig. 4a, b). These results suggest that different regulatory systems may function dependent on the temperature shift conditions. Additionally, in the cold acclimation test involving transfer to 15 °C for 5 h after 25 °C cultivation, N2 and AB1 acclimated to the new temperature faster than the other strains, including CB4856 from Hawaii. These data indicate that a fast rate of cold acclimation is the minority phenotype and that slow cold acclimation, taking over 8 h, is the phenotype of the majority of strains. Habitat and climatic conditions do not appear to have significant correlation with cold acclimation rate or cold tolerance.

### Utilization of whole genome sequences

In this study, we decoded or re-sequenced whole genome sequences of seven wild-type isolates, with coverage of more than 90 % for N2, CB4854, KR314, RC301, AB1, and CB4852, and about 30 % for CB4853 (Supplementary Data 1, 2). To map polymorphisms responsible for causing rapid cold acclimation in the AB1 strain, we compared the sequences of AB1 and CB4856 and created a list of restriction fragment length polymorphisms (RFLPs) between AB1 and CB4856 (Supplementary Data 4). The RFLP list consists of SNPs inducing high and moderate effects. In total, comparing AB1 against CB4856, we identified 6400 RFLP sites, indicating that about one thousand RFLPs per chromosome are available.

### Genetic mapping of natural variation of cold acclimation: multiple polymorphisms independently contribute to rapid cold acclimation in AB1 strain

We used the natural isolate strains AB1 and CB4856 to identify genes responsible for variations in cold acclimation rate (Fig. 4b). Although CB4855, RC301, and other strains showed a phenotype of significantly slow cold acclimation, we did not use these strains for genetic analysis because the survival rates at 2 °C of animals initially cultivated at 25 °C did not recover to high levels until they were cultivated for 8 h at 15 °C prior to the 2 °C incubation (Fig. 4b).

From the results of genetic mapping of the polymorphisms responsible for rapid cold acclimation in the AB1 strain, we have so far identified two genomic regions in which genes involved in cold acclimation are located. The polymorphisms responsible for raising the rate of cold acclimation in KHR074 or KHR075 are probably different because KHR076, which retained the AB1 genome only in their overlapping region, did not show the rapid cold acclimation phenotype. In addition, KHR060 showed slow cold acclimation but retained the AB1-type genome between −1.9 and 2.4 cM, leading to the conclusion that the region between −1.9 and 2.4 cM did not contribute to accelerated cold acclimation in the AB1 strain. Therefore, we need further analysis to identify multiple independent polymorphisms of KHR074 and KHR075. In the region between 1.1 and 5.1 cM on chromosome I, approximately 252 genes were affected by high- and moderate effect polymorphisms. Genes that we previously identified as being involved in cold tolerance (e.g., the insulin receptor *daf*-*2*) are not present in this region. We speculate that unidentified molecular mechanisms of cold acclimation will be found through the identification of the genes responsible for these natural variants.

How the plasticity of cold acclimation is controlled at molecular and cellular levels are important questions. Our previous studies demonstrated that cold tolerance is regulated in at least the nervous system and intestine, whereby temperature is received by a temperature-sensing neuron that then regulates the intestine through insulin (Ohta et al. 2014). As a result, insulin signaling modulates the fatty acid composition in the body (Ohta et al. 2014). Cold acclimation is potentially controlled by the temporal regulation of the strength of these signaling pathways, e.g., the quantity of insulin secreted from a synapse, the plasticity of temperature sensation in neurons, or changing the fatty acid composition. The genes that we previously identified as involved in cold tolerance, such as the insulin receptor *daf*-*2* is not present in this region. Identification of genes involved in cold acclimation from the analysis of natural variations such as AB1 and CB4856 could lead to novel molecular and physiological insights into the plasticity of temperature acclimation.

## Conclusions

Temperature tolerance and acclimation are essential for the survival and proliferation of organisms against environmental temperature changes. In this study, comparative phenotypic analysis revealed that *C. elegans* strains display natural variation in cold tolerance and acclimation rates. A combination of whole genome DNA sequencing and classical positional mapping was helpful to reveal that genes responsible for variation in cold tolerance and acclimation are mapped onto specific chromosomal regions. Future genetic analysis may shed light on a fundamental molecular mechanism for temperature tolerance and acclimation. In addition, genome sequence information of natural isolates will be valuable for comparative genomics and for the identification of genes responsible for diverse phenotypes.

## Electronic supplementary material

Below is the link to the electronic supplementary material.

Supplementary material 1 **Summary and called polymorphisms from whole genome sequencing analysis for KR314, RC301 and CB4854** (XLSX 292 kb)

Supplementary material 2 **Summary and called polymorphisms from whole genome sequencing analysis for AB1, CB4852 and CB4853** (XLSX 203 kb)

Supplementary material 3 **Summary and called polymorphisms for comparison between AB1 and CB4856** (XLSX 2004 kb)

Supplementary material 4 **Restriction fragment length polymorphism (RFLP) sites for distinguishing between AB1 and CB4856.** RFLP sites were identified comparing between AB1 and CB4856, as CB4856 sequence was used as the reference in the first sheet, and AB1 sequence was used as the reference in the second sheet. (XLSX 3027 kb)

Supplementary material 5 (DOCX 595 kb) **Supplementary Figures and Tables**

## Abbreviations

*C. elegans*: *Caenorhabditis elegans*
Indel: Insertion and deletion
SNP: Single nucleotide polymorphism
NGS: Next generation sequencing
NGM: Nematode growth medium
GATK: Genotyper in the Genome Analysis Toolkit
RI: Recombinant inbred
RFLP: Restriction fragment length polymorphism
