# Supplementary material for: Natural variations of cold tolerance and temperature acclimation in Caenorhabditis elegans
Source: J Comp Physiol B. 2016 Jun 18;186(8):985–98. doi: 10.1007/s00360-016-1011-3 (PMC5090014; doi:10.1007/s00360-016-1011-3)
Supplement: Supplementary file 5 — Supplementary material 5 (DOCX 595 kb) Supplementary Figures and Tables [file 360_2016_1011_MOESM5_ESM.docx]

Supplementary Table 1

**Summary of polymorphisms called by comparing AB1 against CB4856.**

The number of polymorphisms called by comparing AB1 with CB4856. The re-sequenced data of CB4856 (Accession Number; SRR443373) was used in this analysis.

Supplementary Table 2

**Distribution of polymorphisms between AB1 and CB4856 among the chromosomes.**

The polymorphisms inducing high and moderate effects (indicated in Supplementary Table 1) were called by comparing AB1 with CB4856, with the CB4856 genome used as reference. The numbers of polymorphisms in this table were counted after removing duplicate polymorphisms present at the same position on each chromosome, because multiple transcriptional isoforms were often occurred by alternative-splicing in the same gene, as shown in Supplementary Table 1.

Supplementary Figure 1

The cold tolerance phenotypes of three natural strain variants, CB4854, RC301, and KR314 were measured when worms were cultured at 17°C and exposed to a cold shock of 2°C for 48 hours. The survival rates of CB4854 and KR314 were 22%, and 60% and were significantly lower than that of the Bristol N2 strain. In contrast, RC301 animals showed a similarly high survival to that of N2. For each assay, n≥9. Error bars indicate SEM. Statistical analyses were performed by one-way analysis of variance for multiple comparisons, followed by Dunnett’s *post-hoc* tests. Asterisks indicate statistical significance between the N2 Bristol strain and the other wild-type isolates **P* < 0.05 and ***P* < 0.01.

Supplementary Figure 2

**Genotypes and phenotypes of representative recombinant inbred lines for identification of the polymorphisms responsible for the CB4854 phenotype.**

Genetic positions on the sex chromosome are indicated as cM. In the genotype columns, N2 and CB4854 indicate the homozygotes of the N2-type and CB4854-type SNPs, respectively. N2/CB4854 indicates the heterozygote of N2 and CB4854 at that position. Blank columns are undefined genotypes by snip-SNPs methods.

Supplementary Figure 3

**The sequence of primers used for genotype analysis.**

(A) This table shows the primers that we used for SNP analysis for mapping of AB1 for responsible genes. We performed snip-SNPs at 1.1 cM, 3.1 cM, 5.1 cM, and 17.3 cM. The other positions, -1.9 cM, 2.4 cM and 2.9 cM SNP were analyzed by direct sequence.

(B) This table shows the primers that we used for SNP analysis for mapping of CB4854 for responsible genes.
